# Comparing output from two methods of participatory design for developing implementation strategies: traditional contextual inquiry vs. rapid crowd sourcing

**DOI:** 10.1186/s13012-022-01220-9

**Published:** 2022-07-19

**Authors:** Emily M. Becker-Haimes, Brinda Ramesh, Jacqueline E. Buck, Heather J. Nuske, Kelly A. Zentgraf, Rebecca E. Stewart, Alison Buttenheim, David S. Mandell

**Affiliations:** 1 Department of Psychiatry, University of Pennsylvania Perelman School of Medicine, 3535 Market Street, 3rd floor, Philadelphia, PA 19104 USA; 2 Hall Mercer Community Mental Health, University of Pennsylvania Health System, Philadelphia, USA; 3 Sandra Rosenbaum School of Social Work, University of Wisconsin—Madison, Madison, WI USA; 4 Department of Family and Community Health, School of Nursing, University of Pennsylvania, Philadelphia, PA USA; 5 Center for Health Incentives and Behavioral Economics, Perelman School of Medicine, University of Pennsylvania, Philadelphia, PA USA

**Keywords:** Participatory design, Evidence-based practice, Implementation science, Implementation strategy design

## Abstract

**Background:**

Participatory design methods are a key component of designing tailored implementation strategies. These methods vary in the resources required to execute and analyze their outputs. No work to date has examined the extent to which the output obtained from different approaches to participatory design varies.

**Methods:**

We concurrently used two separate participatory design methods: (1) field observations and qualitative interviews (i.e., traditional contextual inquiry) and (2) rapid crowd sourcing (an innovation tournament). Our goal was to generate and compare information to tailor implementation strategies to increase the use of evidence-based data collection practices among one-to-one aides working with children with autism. Each method was executed and analyzed by study team members blinded to the output of the other method. We estimated the personnel time and monetary costs associated with each method to further facilitate comparison.

**Results:**

Observations and interviews generated nearly double the number of implementation strategies (*n* = 26) than did the innovation tournament (*n* = 14). When strategies were classified into implementation strategies from the Expert Recommendations for Implementing Change (ERIC) taxonomy, there was considerable overlap in the content of identified strategies. However, strategies derived from observations and interviews were more specific than those from the innovation tournament. Five strategies (13%) reflected content unique to observations and interviews and 3 (8%) strategies were unique to the innovation tournament. Only observations and interviews identified implementation strategies related to adapting and tailoring to context; only the innovation tournament identified implementation strategies that used incentives. Observations and interviews required more than three times the personnel hours than the innovation tournament, but the innovation tournament was more costly overall due to the technological platform used.

**Conclusions:**

There was substantial overlap in content derived from observations and interviews and the innovation tournament, although there was greater specificity in the findings from observations and interviews. However, the innovation tournament yielded unique information. To select the best participatory design approach to inform implementation strategy design for a particular context, researchers should carefully consider unique advantages of each method and weigh the resources available to invest in the process.

Contributions to the literature

- This study advances our understanding of the strengths and limitations of different approaches to participatory design to inform implementation strategy generation.
- The two methods of participatory design—traditional contextual inquiry and an innovation tournament—yielded substantial overlap in the information obtained, but there was unique output generated by each method.
- The innovation tournament required less time investment to complete; however, its electronic platform was expensive, making its overall cost higher than that of observations and interviews
- Results can guide researchers to carefully consider what each method offers and weigh the resources available (e.g., personnel time vs. money) to invest in the process.

## Comparing output from two methods of participatory design for developing implementation strategies: traditional contextual inquiry vs. rapid crowd sourcing

Implementation strategies, the methods used to improve the use and sustainment of evidence-based practices (EBPs), are the backbone of implementation science [1]. Carefully selecting implementation strategies that target theorized implementation mechanisms [2], guided by contextual inquiry to identify implementation determinants [3], is de rigueur for implementation efforts. There are several published methods for selecting and tailoring implementation strategies [2], all of which emphasize the importance both of collaborating with stakeholders, or “end-users,” of the intervention being implemented, and of drawing on methods from participatory design to do so. Because implementation is context dependent, implementation strategies will be more successful if those leading implementation efforts learn from practitioners who will use the intervention of interest. Outputs from contextual inquiry ideally identify implementation determinants, which then drive implementation strategy selection [4, 5].

Implementation science has borrowed from other disciplines to add participatory tools to inform the design of implementation strategies. Participatory design approaches have a long history in many fields and aim to involve core stakeholders directly in co-designing tools, products, and other processes. Participatory design originated as a collaborative method of product design that engages end-users in designing new products using a variety of different methodologies [6, 7]. Participatory design methods can include observation of product use in the field, interviews, and focus groups, as well as traditional user-centered design practices such as obtaining reflective feedback from stakeholders on various mock-ups or prototypes throughout the design phase [6, 8]. Primarily originating in the information technology space [7], the adoption of participatory design methods for designing implementation strategies is more recent.

Most approaches to participatory design in implementation science (e.g., concept mapping, implementation mapping) rely heavily on inputs obtained through rigorous contextual inquiry, using methods such as field observations and qualitative interviews [9, 10]. However, these approaches to participatory design are time intensive and costly. It is also not clear which participatory design approach an implementation scientist should pick when engaging end-users in implementation strategy development for a target EBP, nor is it known whether different approaches will lead to varying results. To our knowledge, no one has compared the results of using different approaches of participatory design on the resultant implementation strategy output [11]. In particular, it would be useful for the field to identify if alternative rapid participatory design methods can produce comparable outcomes to the more traditional methods that can take months to execute and analyze.

To this end, this study compared output from two participatory design approaches: [1] a contextual inquiry approach (i.e., field observations and qualitative interviews with stakeholders) versus [2] a more recent addition to the panoply of participatory design tools, an innovation tournament, which is intended as a more rapid approach to gaining end-user feedback to inform implementation strategy design. Innovation tournaments involve a kind of crowd sourcing—a competition in which members of a group (in this case, relevant stakeholders to an implementation problem) can submit ideas about how to address a challenge. These ideas compete with one another through several rounds of vetting. Other members of the group can vote for and comment on participants’ different ideas (thus engaging in the reflective practice from stakeholders that is integral to all participatory design methods; 6). In the last round, experts work with the originators of the most popular and promising ideas to refine them for a last round of voting. Innovation tournaments have become particularly popular in health care and other industries [12–14]. Stewart and colleagues recently used one to identify potential implementation strategies to increase the use of evidence-based practices in outpatient mental healthcare, demonstrating their feasibility and potential utility in generating tailored implementation strategies in behavioral health [15]. As innovation tournaments are designed to quicker, cheaper, and less burdensome on stakeholder partners, identifying whether their output produces comparable results to those of more traditional contextual inquiry approaches has important implications for how we use participatory design in implementation science.

Our secondary aim was to categorize the resources involved with executing and analyzing the data obtained from each participatory design approach. Given the breadth of potential implementation strategies that emerged from Stewart and colleagues’ innovation tournament, we hypothesized the innovation tournament would produce output comparable to that obtained from observations and qualitative interviews, with less burden on stakeholders and research staff.

## Methods

This work was conducted as an exploratory project as part of the Penn ALACRITY Center which aims to integrate behavioral economics and implementation science principles to increase evidence-based practice use [16]. In this exploratory project, we aimed to employ principles of participatory and user-centered design to design a mobile application to support data collection among behavioral health technicians (BHTs) who work one-on-one with children with an autism spectrum disorder in schools [17]. Quantitative data collection on child response to intervention and progress monitoring is a core EBP component of applied behavioral analysis (ABA) for children with autism yet is poorly implemented in practice [18]. As formative work in the design and development of this app, and in keeping with the goals of the Penn ALACRITY Center to test new methods of participatory design, our team concurrently [1] collected observations and interviews with BHTs and interviews with their supervisors and [2] conducted an innovation tournament inviting BHTs and their supervisors, to participate. We synthesized data obtained from each method using a systematic, iterative processes to identify implementation strategies.

To avoid cross-contamination of output from each method, the research team divided into two separate groups for the duration of data collection and synthesis so that each team was blinded to outputs or information obtained from the other method of participatory design. We briefly describe the participatory design strategy and data analysis below. The City of Philadelphia (Approval #2019-32) and University Pennsylvania (Approval #849995) Institutional Review Boards approved the study.

### Observations and interviews

We recruited 9 BHTs and 7 BHT supervisors from four different agencies in Philadelphia (63% female; 31% White; 44% Black; 6% Asian; 19% Hispanic/Latinx). Recruited BHTs had to be working with a school-aged child with autism. BHTs allowed us to observe their data collection practices in the field and participated in a subsequent qualitative interview over the phone. Two BHTs completed a qualitative interview only due to COVID-19 social distancing restrictions put into place in the final month of planned data collection. Recruited BHT supervisors participated in phone interviews only. To protect BHT participant confidentiality, BHT supervisors were recruited separately from participating agencies, rather than as matched BHT/BHT supervisor pairs. We obtained informed consent from each participant prior to any research activities.

Prior to observations, author HJN trained research team members in a structured observation coding system adapted to document barriers and facilitators to BHT data collection [19, 20]. Coders were trained to identify opportunities for data collection on the part of BHTs across 3-min intervals, whether or not the BHTs engaged in data collection on available opportunities and take field notes on observed barriers and facilitators to data collection.^1^ After obtaining BHT consent and formal approval from the school in which the BHT worked, research staff traveled to the BHT’s work site for a 1-h field observation. Two research team members (EBH and BR) attended the first two observations to ensure reliable use of the structured field observation form; one team member completed the remaining observations and debriefed with the second team member immediately after. During each observation, research staff observed BHT data collection practices from the back of the classroom using the structured form. Research staff noted, in real time, perceived barriers to data collection they observed. At the conclusion of the 1-h observation, staff spent approximately 5 min asking the BHT to expand on observed barriers (e.g., “I noticed you didn’t take data in X interval. What, if anything, got in the way of you doing so?”). After the observation, BHTs scheduled a qualitative interview.

Qualitative interview guides for BHTs and BHT supervisors were designed to assess data practices BHTs used, assess barriers to engaging in quantitative data collection, and elicit recommendations on how quantitative data collection could be made easier for BHTs. BHTs additionally answered standard belief elicitation questions and questions adapted from a theory-guided interview created by Potthoff and colleagues [21, 22]. Copies of the interview guides are available upon request. Team members met and reviewed emerging themes following every 2–3 interviews; when two successive interviews did not yield any new substantive information across both stakeholders, we determined that saturation was reached. Thematic saturation was reached after the 9^th^ BHT interview and the 7th BHT supervisor interview.

The interviewer audio-recorded the interview and a member of the research team transcribed the recording. The researchers who conducted the field observation (EBH or BR) analyzed the interviews, so that information learned via observations could guide interpretation of qualitative responses. The research team developed a structured codebook via iterative review of three transcripts and applied it to the remaining transcripts. The a priori code of “barriers and facilitators to quantitative data collection” was the primary code of interest for this study. Coders met regularly to review codes and resolve discrepancies.

From the coded transcripts, we amassed a list of 10 identified major barriers across BHTs and BHT supervisors. The same two staff members systematically reviewed each identified barrier and generated one or more discrete implementation strategies that would address each barrier drawing on their review of all qualitative data and field observation notes collaboratively and systematically, guided by leading frameworks to guide implementation strategy design: the Expert Recommendations for Implementing Change (ERIC) taxonomy and the Behavior Change Wheel [23, 24]. To ensure comprehensiveness, a third team member who had administered qualitative interviews but was not involved in the coding process (KAZ) then reviewed the list of barriers and independently generated corresponding implementation strategies. This third team member remained masked to the original generated implementation strategies prior to a consensus meeting and did not identify any additional implementation strategies. This process resulted in a final set of 26 discrete implementation strategies, with two to five strategies generated per barrier (some generated strategies also addressed multiple barriers).

### Innovation tournament

Our innovation tournament procedure was guided by Stewart and colleagues, which was developed through the Penn Medicine Center for Health Care Innovation [13, 15]. We invited behavioral health professionals working with children with autism in Philadelphia schools (*n* = 21), including BHTs and their supervisors, who had provided us their email addresses at previous in-person site visits. We sent them email invitations to participate in an innovation tournament titled “Your Idea Matters! The Data Collection Improvement Challenge.” We also asked behavioral health agency leaders (*n* = 27) and team meeting leaders at in-person site visits (*n* = 5) to forward recruitment invitations to their staff. Some BHTs may have received the invitation more than once (i.e., from a direct email from our team, from their agency leader, and from their team meeting leader). We sent agency leaders a priming email approximately 2 weeks prior to the start of the tournament, as advance contact aligns with recommendations in survey design [25]. We then sent the first invitation email to agency leaders, team meeting leaders, and BHTs, which included the link to participate, and 5 reminder emails over the course of 5 weeks. During the second week of reminders, we contacted agency leaders to participate and invited their staff to participate by phone.

We made the web link to the innovation tournament available for 5 weeks in October 2019. Participants opened the link and provided consent before participating. We hosted the online tournament on the “Your Big Idea Platform” that facilitates crowdsourcing for ideas and solutions. Innovation challenges are posted to the platform, and participants can submit an idea and comment and rate ideas submitted by other participants. As such, the electronic platform supports both the submission of ideas as well as hosting the full list of suggestions made for participants to review, rate, and vote for the suggestions they think represent the best solutions for the challenge problem.

Developing the right tournament prompt (i.e., the question for participants) is critical to the success of an innovation tournament [13, 15]. We iteratively designed the prompt for this study. The research team generated the question language via email and in weekly meetings and then sought feedback from 2 BHTs on the wording and design of the prompt. The final prompt asked, “What would make data collection about children with autism easier, more useful, and more motivating for you?” Participants could submit and rate as many ideas as they liked.

Eleven individuals submitted 14 ideas. Procedures for reviewing, rating, identifying “winning ideas,” and categorizing output followed established procedures [15]. Briefly, the research team formed a Challenge Committee consisting of an expert panel of stakeholders to vote on the submissions. The committee included two city administrators, two agency stakeholders, one autism content expert, and one behavioral science expert. The committee rated the 14 ideas on their potential impact as an implementation strategy. Participants with the top three ideas were considered “winning participants;” these participants were later celebrated at a community event intended to foster community engagement in the implementation of evidence-based practices for autism.

To categorize information shared through the innovation tournament platform into clear implementation strategies, three members of the research team (JEB, RES, DSM) met and applied aspects of the NUDGE (Narrow, Understand, Discover, Generate, Evaluate) framework to extrapolate the discrete implementation strategies from the information submitted by innovation tournament participants. See [26] for a detailed description of how the NUDGE framework is applied to analysis of innovation tournament data. Briefly, this is an iterative process of: [1] generating hypotheses about behavioral barriers that interfere with EBP delivery through structured brainstorming that links the reported barriers to core principles from behavioral science (e.g., cognitive biases and heuristic thinking), [2] de-duplicating hypotheses, and [3] rapidly validating hypothesized barriers through expert consultation, review of the literature, and confirmation by core stakeholders. This output then informs generation of tailored implementation strategies to directly address the behavioral barriers identified. We selected the NUDGE framework to guide generation of implementation strategies here as it aligned with methods of the only other innovation tournament used in mental health care to date [15, 26].

### Resources and cost of each method

We captured the time and costs associated with each method, guided by principles of time-driven activity-based costing, a method increasingly employed in implementation science [27]. Researchers who executed each participatory design method estimated the time and money spent throughout the process of data collection and synthesis.

## Results

After output from each method was used to generate implementation strategies, guided by each method’s respective analytic framework (i.e., the NUDGE framework for the innovation tournament and the ERIC and the Behavior Change Wheel for the traditional contextual inquiry), we then categorized each implementation strategy using the ERIC taxonomy to facilitate comparison of output content. Table 1 shows the implementation strategies that the two participatory design approaches generated. The first author (EBH) classified all implementation strategies across the two approaches using the ERIC taxonomy, using the clusters identified by Waltz and colleagues [24, 28]. An independent reviewer (HJN) was trained in the ERIC taxonomy and then double coded 50% of all strategies, with 100% concordance achieved without the need for a consensus procedure. To further facilitate comparison, we also classified how, within each ERIC cluster, the implementation strategy generated from each method compared when examined at sub-clusters identified by Powell and colleagues 2015 within the ERIC taxonomy [29].

**Table 1:** Output from innovation tournament compared with observations/qualitative interviews

		Identified implementation strategies	
Implementation strategy cluster^a^	Implementation strategy^b^	Innovation tournament (***n*** = 14 strategies)	Observations/qualitative interviews (***n*** = 26 strategies)
*Adapt and tailor to context*	*Promote adaptability*	*None*	1. Adapt data collection processes to make them easier to do within the chaotic school environment^d^ 2. Ensure that data required for tracking is of behaviors that can reasonably be tracked from a short distance^d^
*Change infrastructure*	*Change physical structure, equipment, and regulatory processes* ^c^	1. Integrate data collection processes into course of day 2. Adjust regulatory process to increase BHT feedback integration into treatment planning	1. Advocate for adapting the timing of treatment plan regulations to link to changes in behavior rather than calendar months 2. Provide supplies to ameliorate the strain caused by heavy laptops and notebooks (e.g., a small standing desk) 3. Reposition students, teachers, and BHTs in classroom to better facilitate intervention and data collection
	*Change record systems*	1. Changes the forms or templates used to collect data 2. Digitize data collection to reduce effort 3. Make forms simple to use 4. Rely on drop down menus and tallies	1. Eliminate Wi-Fi dependency of electronic data record 2. Consult with software developers to address limitations of current data collection capture systems in the electronic health record 3. Eliminate redundancies in data collection processes by eliminating paper forms and conducting direct data entry into the required electronic health record 4. Redesign data capture software to allow for the flexibility required to capture the range of data for specific client 5. Utilize mobile electronic data tracking platform that builds in time clocks to facilitate monitoring across environments or supply all BHTs with watches 6. Ensure that mobile electronic data tracking platforms are light and easy to transport
*Engage consumers*		*None*	*None*
*Evaluative and iterative strategies*	*Conduct local needs assessment*	*None*	1. Assess the strengths and limitations of current data collection system^d^
	*Audit and feedback*	1. Give feedback to BHTs on treatment plans^d^	*None*
*Provide interactive assistance*	*Provide clinical supervision*	1. Offer in vivo coaching 2. Provide immediate feedback on data collection	1. Provide on-site coaching to BHTs on how to balance data with intervention in response to challenging behaviors 2. Enhance channels of communication between BHTs and supervisors 3. Increase the amount of supervisory support available to BHTs, particularly as it relates to direct observation in the field 4. Hold regular supervision meetings in which the BHT and supervisor troubleshoot around limitations of current data collection system^d^ 5. Hold regular supervision meetings in which the BHT discusses examples of competing demands and/or role-plays data collection during problem behaviors
*Support clinicians*	*Facilitate relay of clinical data to providers*	1. Show the BHTs how their data is used with visuals	1. Enhance perceived utility of data collection (e.g., through feedback of client progress)
	*Remind clinicians*	*None*	1. Provide regular reminder mechanism for clinician, e.g., hourly text message^d^ 2. Include definitions of target behaviors for data collection on data record sheet^d^
*Training and educate stakeholders*	*Conduct educational meetings*	1. Improve training 2. Provide skills training in functional analysis	3. Conduct best data collection practices training for active and outdoor times 4. Conduct training on best practices for data collection while clients display problem behaviors 5. Provide portable educational materials to remind BHTs on best practices for collecting data on client problem behaviors 6. Provide targeted skills training to BHTs on how to balance data collection with intervention in response to challenging behaviors 7. Provide targeted training for BHTs in defining behaviors for data collection 8. Train BHTs in data collection interface, including each feature’s function and use
*Utilize incentive strategies*	*Alter incentive/allowance structures*	1. Acknowledge BHT for collecting data^d^ 2. Increase rewards for data collection^d^	*None*

^a^Clusters from Waltz and colleagues 2015

^b^Strategies from Powell and colleagues 2015

^c^This domain was expanded from the traditional “Change Physical Structure and Equipment” to additionally encompass regulatory infrastructure

^d^Implementation strategy content not reflected in output from the opposing method

As seen in Table 1, observations and interviews resulted in nearly double the number of implementation strategies (*n* = 26) than did the innovation tournament (*n* = 14). Together, both methods identified implementation strategies that spanned eight of the nine clusters of implementation strategies identified by Waltz and colleagues [26]. Only the observations and interviews generated implementation strategies in the cluster of strategies related to “adapting and tailoring to context” and only the innovation tournament generated implementation strategies in the cluster of strategies related to “utilize incentive strategies.” No implementation strategies from either method were generated in the cluster of engaging consumers (e.g., involving children and their families).

At the more granular level, resultant strategies across both methods spanned 10 discrete implementation strategy categories from the ERIC taxonomy. Although the observations and interviews generated more strategies than the innovation tournament did, a substantial portion of the output from observation and interviews overlapped conceptually with that from the innovation tournament data, albeit with a greater level of specificity emerging from observations and interviews. Specifically, when examined by implementation strategy category, 32 of the 40 total identified implementation strategies (80%) reflected overlapping content obtained from each method. Areas of overlap at this level of implementation strategy category were observed for changing the physical structure, equipment and regulatory processes (*n* = 2 strategies from innovation tournament, *n* = 3 strategies from observations/interviews), changing record systems (*n* = 4 strategies from innovation tournament, *n* = 6 strategies from observations/interviews), providing clinical supervision (*n* = 2 strategies from innovation tournament, *n* = 5 strategies from observations/interviews), facilitating relay of clinical data to providers (*n* = 1 strategy from innovation tournament, *n* = 1 strategy from observations/interviews), and conducting educational meetings (*n* = 2 strategies from innovation tournament, *n* = 6 strategies from observations/interviews). With respect to areas of conceptual divergence between the two methods, only the observations and interviews yielded implementation strategies in the categories of promoting adaptability (*n* = 2 strategies), conducting local needs assessment (*n* = 1 strategy), and reminding clinicians (*n* = 2 strategies). Only the innovation tournament yielded implementation strategies in the categories of: audit and feedback (*n* = 1 strategy), and altering incentive/allowance structures (*n* = 2 strategies).

As noted above, within each of these areas, findings from the observations and interviews tended to yield more specific and somewhat more actionable implementation recommendations than the innovation tournament did. For example, the innovation tournament identified the importance of “improving training” and “providing skills training in functional analysis.” In contrast, observation and interview data yielded 6 specific recommendations to improve training in response to specific implementation barriers to data collection that BHTs reported. For example, specific strategies included training on best data collection practices, training for active and outdoor times, and training on best practices for data collection when clients display problem behaviors. Similarly, for “provide clinical supervision,” the innovation tournament indicated the importance of offering in vivo coaching and providing immediate feedback on data collection. In contrast, the observation and interview data yielded 5 more specific recommendations about how supervisory processes could better address specific barriers to evidence-based data collection for BHTs.

Table 2 shows the estimated costs of each participatory design method, separated by preparation costs, data collection costs, and data synthesis costs. Overall, personnel time was substantially higher for the observations and interviews (~ 263 h) than for the innovation tournament (~ 68 h). However, the overall cost of the innovation tournament, which relied on an electronic platform to power idea submission and allow participants to comment on and rate ideas submitted by other participants, was much higher than that for observations and interviews.

**Table 2:** Estimated costs associated with each participatory design approach

	Innovation tournament			Observations/qualitative interviews		
	Activity	Personnel time (hours)	Cost ($)	Activity	Personnel time (hours)^a^	Cost ($)
***Preparation***						
	Generate innovation tournament prompt	10	200	Develop recruitment materials	12	240
	Set up “Your Big Idea” platform	10	200	Recruitment outreach (e.g., agency visits, travel costs, communication with potential participants)	32	720
	Recruitment outreach (e.g., phone calls, emails)	10	200	Obtain letters of support (includes travel to sites and car rental fee)	20	426
				Training in observation and interview procedures	20	400
***Total time and cost for preparation***		**30**	**600**		**84**	**1786**
***Data collection***						
	Operating innovation tournament on the “Your Big Idea Platform”	12	14,594	Conduct observations (*n* = 7)	42	840
	Innovation tournament activities (notify winners, set up meetings, discuss ideas)	10	200	Conduct interviews (*n* = 17)	18	160
				Interview transcription	60	1200
***Total time and cost for data collection***		**22**	**14,794**		**120**	**2200**
***Data synthesis***						
	Behavioral diagnostics data analysis	16	320	Develop and train on qualitative coding guide	21	420
				Code interviews	20	400
				Analyze barriers and generate implementation strategies	18	360
***Total time and cost for data synthesis***		**16**	**320**		**59**	**1180**
***Total overall cost***		**68**	**15,714**		**263**	**5166**

^a^Some tasks involved more than one person; in these instances, personnel time was multiplied accordingly. Cost was calculated based on a rate of $20/hour/person across all activities to facilitate comparability across the two methods

## Discussion

To our knowledge, this is the first effort to directly compare the output of two distinct methods of participatory design and their resultant implications for implementation strategy design. We compared the output of two different participatory methodological approaches—an innovation tournament and observations and interviews. Data suggested a high degree of convergence in output across the two strategies when examining concordance at the broadest conceptual levels (i.e., within the general theoretical clusters of implementation strategies). However, the information yielded by the traditional contextual inquiry was much richer and detailed than that obtained from the innovation tournament. Overall, the observations and interviews generated a greater number of recommendations and more specific recommendations than did the innovation tournament. Given that a growing body of literature highlights the importance of tailored implementation strategies to lead to optimal implementation outcomes [2, 4], we might hypothesize that the more specific implementation strategies generated by the traditional contextual inquiry may be more effective than the more general strategies generated by the innovation tournament. However, the contextual inquiry took almost four times as long. That said, each method has strengths and limitations that might inform a decision to use one method or another. Table 3 presents a high-level overview of the strengths and limitations of each method studied to guide researchers’ decisions about how and when to use one method over another.

**Table 3:** Strengths and limitations of each method

	Innovation Tournament		Observations/Qualitative Interviews	
	Strengths	Limitations	Strengths	Limitations
***Preparation phase***	• Only need to create a single prompt	• Limited to a single prompt to elicit information about potentially complex problems	• Have option to ask a range of questions to inform implementation strategy design	• Time and resource intensive (both with creating materials and training and supervising research staff)
***Data collection and synthesis phase***	• Limited time burden placed on stakeholders (stakeholder participation time is low, can participate when and where they choose) • Data analysis less time intensive than traditional qualitative interviews • Stakeholder voice is involved in analysis through voting/vetting of ideas	• Cannot iteratively refine prompts based on initial responses from stakeholders • “Stopping” the tournament not traditionally linked to reaching thematic saturation	• Can continue to refine questions over time as new information is gathered • Can determine “stopping” point based on achieving thematic saturation	• Time and resource intensive (both with regards to data collection and training and supervising of research staff)
***Community engagement***	• Iterative “voting” process during the data collection phase intended to create community and buy-in among stakeholders • Unlimited number of participants can share ideas • Low incremental cost to adding more participants	• Difficult to engage individuals who may be less likely to be engaged via electronic medium	• Participants can identify other core stakeholders to be included • Can identify key individuals to serve on an advisory board	• Engagement is with a subset of stakeholders only • High incremental cost of adding more stakeholders to process
***Overall***	• Ideal for a specific question with potentially straightforward solutions • Requires fewer person hours • Lower stakeholder burden • Results can be analyzed quickly with low person power	• Less detailed information about context, leading to less targeted implementation strategy suggestions • Electronic platform can be costly	• Provides greater detailed insight into context, informing more targeted implementation strategy suggestions	• Greater burden placed on stakeholders • More time and person power required to complete all phases

While our results do suggest that observations and interviews overall resulted in more specific implementation strategies than did the innovation tournament, there was still unique information produced by each. As such, results from this study do not suggest a clear recommendation that one participatory design method is clearly superior to the other. The time and resource element should be given significant consideration. Overly long contextual inquiry efforts to design implementation strategies may further delay the integration of evidence into practice or providing stakeholders with critical feedback during the implementation. It remains an empirical question how to trade off efficiency in contextual inquiry and detail in implementation strategy output to optimize acceleration of research into practice. Researchers should also carefully weigh the potential information gaps that may occur from using one participatory design method and consider stakeholder preferences for implementation strategies. For example, contextual inquiry was better at generating strategies relating to adapting and tailoring interventions to context, whereas the innovation tournament generated more strategies related to incentives, the latter being considered “most useful” by clinicians, supervisors, and payers [30]. Surprisingly, no implementation strategies from either method were generated in the cluster of engaging consumers, and perhaps direct prompts are needed to generate strategies that include this important stakeholder group. Future work could explore how integrating methods, such as conducting an initial innovation tournament followed by rapid stakeholder feedback (e.g., “member checking”), could perhaps lead to greater efficiency in the contextual inquiry process than more traditional methods. For example, innovation tournament output could be reviewed briefly with local champions to delineate the specific suggestions that emerged from the formal contextual inquiry. This would be an important area for future research to determine if comparable specificity of information could be obtained in a more expedient fashion through an innovation tournament compared to interviews and observations.

It is also important to note that while we might hypothesize that the more specific strategies generated by observations and interviews might be more effective than the more general ones generated by the innovation tournament, our data are not able to determine whether the output of one participatory design method is more effective, acceptable, or feasible than another. An important next step in this line of research will be to share the resultant strategies from each method with stakeholders and evaluate their perception of each strategy’s acceptability and feasibility. Whether stakeholder perceptions vary as a function of the chosen participatory design approach it was derived from is an important question to explore in future research.

Ours findings suggested clear limitations of innovation tournaments as compared to more traditional contextual inquiry. That said, the innovation tournament did yield both somewhat comparable and unique information (related to incentives) to that obtained from the observations and interviews and required substantially less time investment from both the research team and stakeholder participants to complete. The primary driver of the cost of the innovation tournament was the electronic platform used in this study. Researchers hoping to conduct an innovation tournament to gain more rapid contextual inquiry could use cheaper HIPAA-compliant survey platforms to elicit ideas from stakeholders in response to an innovation prompt. However, to include rounds of coding and stakeholder involvement in rating of ideas, along with various incentives for the “winners’ of the innovation tournament, would be more costly. Should costs for such technology reduce in the future, innovation tournaments may hold potential to be a more cost-efficient way to gather rapid information from stakeholders and should be continued to be studied as an alternative method to engage with stakeholders to address implementation challenges.

There are several reasons why output may differ between the two methods we studied. Observations and interviews allowed for the “up close and personal” examination of the BHT workflow and environment, as well as opportunities for follow-up questions; this may have facilitated obtaining information about adapting and tailoring to context that did not emerge from the innovation tournament. In contrast, the innovation tournament may have been more likely to identify incentive-related strategies because the prompt allowed for emotional “distancing” on the topic from respondents. Specifically, innovation tournament participants were asked to report on what would make data collection for BHTs easier and more motivating broadly (in contrast to speaking specifically to the barriers and facilitators they faced in data collection in the qualitative interview). Participants may have been more likely to think that their colleagues would be motivated by rewards or other incentives to engage in data collection, even if they perceived that they had their own intrinsic motivation to do so, and that the innovation tournament was more likely to elicit this type of information. Participants also may have felt more comfortable sharing ideas about monetary rewards in a more anonymous setting like the innovation tournament, rather than face-to-face during interviews.

Finally, it is possible that the fundamentally different approach to the framing of the inquiry in each approach led to the divergence in findings. During the qualitative interviews, research staff asked open-ended questions barriers and facilitators to data collection and then deduced potential solutions to the information shared in the generation of implementation strategies. In contrast, the innovation tournament asked respondents to report directly on suggested solutions to the challenge of how to make data collection easier and more motivating and then the research team refined the offered strategies. Therefore, in many ways, the implementation strategies generated from the observations and interviews incorporate the researchers’ experience and expertise in a way that the innovation tournament does not.

There are also likely common disadvantages to both methods that are important to note. In general, people tend to be inaccurate reporters on what would change their own behavior [31]. Outputs from both methods may suffer from biased sampling, as all data comes from participants amenable to collaborating with researchers. This may have contributed to the lack of implementation strategies from both methods related to engaging consumers; had we included youth and their families in each participatory design process, results may have differed.

We note several study limitations. First, for both participatory design methods, we had relatively small samples sizes. While our observations and interviews reached thematic saturation, our sample for the innovation tournament was lower than others’ [13]. BHTs comprise a largely independent contractor workforce, and they often work independently at schools, which may have contributed to our difficulty engaging them. In addition, the innovation tournament is not necessarily designed for concluding when saturation is reached, as there is limited control over sample size. Second, we did not evaluate the acceptability, feasibility, or effectiveness of any of the identified implementation strategies, so we are unable to evaluate the fit or effectiveness of each participatory design approach, only to highlight points of convergence and divergence. Third, we employed a different framework to generate implementation strategies in the innovation tournament (i.e., the NUDGE framework) than within the traditional contextual inquiry (i.e., ERIC/Behavior Change Wheel for). While our rationale for using the NUDGE framework was to replicate methods of the only other published innovation tournament for mental healthcare, it is possible that this decision may have led to additional divergence in outcomes between the innovation tournament and traditional contextual inquiry. Fourth, as noted above, we did not interview youth and families in this study, nor did we invite them to participate in the innovation tournament, which may have led to a gap in identified implementation strategies. This underscores the potential for exclusion of select stakeholder groups to greatly impact the output of a participatory design approach. Finally, it remains an open question whether one might find the same points of overlap and divergence in output between observations and interviews with an innovation tournament when conducted in another setting (e.g., within a large healthcare system or with providers outside of the mental health space). Our hope is that proposed strengths and limitations of each method delineated in Table 3 can help support researchers working in other healthcare spaces to determine which method will best address their implementation questions; however, this remains a question for future research.

This study also has some notable strengths. Our use of two separate and blinded research teams to execute each participatory design method concurrently allowed us to be confident in the independence of the findings from each method. Importantly, this study represents the first effort to compare the results that emerge from two methods of participatory design, setting the stage for future research to refine selection and execution of participatory methods to optimize the design of effective implementation strategies.

## Conclusions

This study indicates that two methods of participatory design—observations/interviews and an innovation tournament—yield substantial overlap in the information obtained that can be used for implementation strategy development. However, in this study, the observations and interviews resulted in more specific and tailored implementation strategies than did the innovation tournament. That said, there was also unique output generated by each. Given the time and resources required to engage in comprehensive contextual inquiry, selecting the best participatory design approach to inform implementation strategies necessitates that researchers carefully consider what each method offers (strategies relating to adapting and tailoring to context vs. incentives) and weigh the resources available (e.g., personnel time vs. money) to invest in the process. This study advances our understanding of the strengths and limitations of different approaches to participatory design, which is critical for helping implementation researchers and policy makers select the approach best suited to address their implementation question.

## Data Availability

Data will be made available upon request. Requests for access to the data can be sent to the corresponding author.

## Abbreviations

EBP: Evidence-based practice
BHT: Behavioral health technician
ABA: Applied behavior analysis

## Footnotes

1 Additional information about the coding system and training procedures are available from the first author upon request.
